# Paralysie obstétricale du plexus brachial (POPB): aspects épidémiologiques, thérapeutiques et évolutifs au Centre Hospitalier Universitaire de Bouaké, Côte d’Ivoire

**DOI:** 10.11604/pamj.2021.38.309.22940

**Published:** 2021-03-26

**Authors:** Célestin Adoubs Bénié, Jean Régis Achié Akobé, Franck Grah Lohourou, Ibrahim Traoré, Jean Bertrand Ahua Kpangni, Natacha Adelaïde Aya Kouassi, Inza Bamba

**Affiliations:** 1 Unité de Chirurgie Pédiatrique du Centre Hospitalier Universitaire de Bouaké, Bouaké, Côte d’Ivoire,; 2 Service d´Orthopédie Traumatologie du Centre Hospitalier Universitaire de Bouaké, Bouaké, Côte d’Ivoire

**Keywords:** Nouveau-né, paralysie obstétricale, plexus brachial, Newborn, obstetric palsy, brachial plexus

## Abstract

**Introduction:**

la paralysie obstétricale du plexus brachial est une affection relativement rare mais qui n´a pas disparu malgré les importants progrès en obstétrique. L´objectif de cette étude était de décrire les aspects épidémiologiques, thérapeutiques et évolutifs de cette affection dans notre contexte.

**Méthodes:**

analyse rétrospective sur deux ans des dossiers de nouveau-nés ayant présenté une paralysie obstétricale du plexus brachial et pris en charge au Centre Hospitalier Universitaire de Bouaké. Les enfants reçus après l´âge de 3 mois n´ont pas été inclus. Les variables étudiées étaient d´ordre épidémiologique, thérapeutique et évolutif.

**Résultats:**

soixante patients ont été colligés, soit une fréquence de 28,5%. Il y avait 31 (52%) filles. L´âge moyen était de 8 jours [J0 et J35]. Les mères étaient multipares dans 94% des cas. L´accouchement a eu lieu dans un centre de santé dans 97% des cas. Tous les enfants étaient nés à terme avec 57 (95%) présentations céphaliques. L´accouchement était eutocique dans 74% des cas. Le poids moyen de naissance était de 3604g [2150g et 4500g]. Il y avait 47 cas (78%) de paralysie C5-C6. L´immobilisation coude au corps associée à la rééducation a été réalisée chez 51 enfants (85%). La rééducation a été réalisée immédiatement chez 9 enfants (15%). La récupération fonctionnelle du membre lésé a été complète chez 50 enfants (83%) après un recul de 6 mois.

**Conclusion:**

la paralysie obstétricale du plexus brachial demeure une affection obstétricale d´actualité. Son traitement conservateur, seule alternative dans notre contexte donne de bons résultats.

## Abstract (en)

**Introduction:**

obstetrical brachial plexus palsy is a relatively rare condition that has not disappeared although important progress has been made in obstetrics. The purpose of this study is to analyze the epidemiological, clinical, therapeutic and evolutionary features of this disorder in our context.

**Methods:**

we conducted a retrospective study of the medical records of newborns with obstetrical brachial plexus palsy treated at the Bouaké University Hospital over a period of two years. Children treated after the age of 3 months weren´t included. The variables studied were the epidemiological, therapeutic and evolutionary features.

**Results:**

the study included 60 patients, reflecting a rate of 28.5%. There were 31 (52%) girls. The average age of patients was 8 days (D0 and D35]. Multiparous mothers accounted for 94% of cases. Birth took place in a health center in 97% of cases. All children were born at term, 57 (95%) in cephalic presentation. Delivery was normal in 74% of cases. Average birthweight was 3604g [2150g and 4500g]. Forty seven cases (78%) had C5-C5-C6 palsies . Immobilization elbow off body associated with rehabilitation was performed in 51 children (85%). Rehabilitation was performed immediately in 9 children (15%). Functional recovery of the injured limb was complete in 50 children (83%) after a follow-up period of 6 months.

**Conclusion:**

obstetrical brachial plexus palsy is a current obstetric disorder. Conservative treatment, that is the only alternative in our context, gives good results.

## Introduction

La paralysie obstétricale du plexus brachial (POPB) est une lésion nerveuse concernant les racines C5 à T1, qui survient au cours de l´accouchement. Elle résulte de la traction traumatique entre la tête et l´épaule lors du passage de la filière génitale au cours de la délivrance fœtale [1-3]. Elle est décrite dans la littérature comme étant un évènement inhabituel, imprévisible et inévitable [4,5]. C´est une lésion relativement rare mais son incidence semble ne pas diminuer, avec un taux variant de 0,05 à 0,26% de tous les accouchements [1,6]. Les facteurs de risque sont notés chez la mère comme chez le nouveau-né. Les principaux sont retrouvés chez le nouveau-né: il s´agit essentiellement de la macrosomie, la dystocie de l´épaule et la présentation de siège [2,7,8]. Le diagnostic de la POPB est clinique avec des atteintes nerveuses variables et graves mises en évidence par l´électromyogramme ou l´imagerie par résonance magnétique (IRM) neurologique [9,10]. Le traitement est fonction du type de POPB. Le traitement conservateur associant immobilisation et rééducation peut permettre une récupération totale des POPB moins sévères. Pour les formes sévères, la chirurgie constitue la principale indication pour espérer une récupération de la fonction du membre. Le pronostic est variable avec souvent des séquelles majeures invalidantes pouvant avoir des implications médicolégales [6,11,12]. Notre unité constitue un centre de référence pour la prise en charge des POPB. Cependant, peu d´études ont été réalisées sur cette affection à Bouaké. Le but de ce travail était de décrire les aspects épidémiologiques, thérapeutiques et évolutifs.

## Méthodes

Il s´agissait d´une étude rétrospective réalisée entre janvier 2017 et décembre 2018 à l´unité de chirurgie pédiatrique du CHU de Bouaké. Les données ont été colligées à partir des dossiers médicaux et des carnets de naissance des nouveau-nés et nourrissons pris en charge pour une POPB. Le diagnostic clinique a été posé sur la base des critères cliniques de la classification de Narakas [13] (Tableau 1). Les dossiers ayant des données manquantes et les enfants reçus après l´âge de 3 mois n´ont pas été inclus. Le traitement a consisté à une immobilisation coude au corps de type Dujarrier avec une bande Velpeau chez tous les nouveau-nés reçus avant l´âge de 3 semaines. A partir de 3 semaines de vie, le bébé était référé en médecine physique pour des séances de rééducation fonctionnelle. Cette rééducation consistait en des séances de stimulation, de Strapping en rotation externe, de stimulation d´éveil musculaire avec pour objectif, l´entretien articulaire et musculaire en attendant la récupération nerveuse. Le nourrisson était ensuite revu en consultation pour une réévaluation à 3 mois et 6 mois. Les aspects épidémiologiques, cliniques et les résultats fonctionnels ont été étudiés. Les résultats fonctionnels ont été jugés sur des critères cliniques de récupération complète, partielle ou l´absence de récupération sans données électromyographiques. Les déformations persistantes après plus de 6 mois de traitement, ont été considérées comme des séquelles.

**Tableau 1:** classification de Narakas

Groupes	Clinique
**Groupe I : atteinte C5-C6**	Adduction de l´épaule, rotation interne de l´épaule, coude en extension
**Groupe II : atteinte C5-C6-C7**	Adduction de l´épaule, rotation interne de l´épaule, coude en extension, poignet tombant
**Groupe III : atteinte C5-C6-C7-C8-T1**	Paralysie complète sans syndrome de Horner: paralysie complète flasque
**Groupe IV: atteinte C5-C6-C7-C8-T1**	Paralysie complète avec syndrome de Horner

## Résultats

Soixante patients ont été colligés, soit une fréquence de 28,5%. Il y avait 31 (52%) filles et 29 (48%) garçons, avec un sex-ratio à 1,06 au dépend du sexe féminin. L´âge moyen à l´admission était de 8 jours [J0 et J35]. Les caractéristiques épidémiologiques des mères et le déroulement de l´accouchement sont répertoriés dans le Tableau 2. La durée du travail était supérieure à 8 heures chez 50 parturientes (83%). L´accouchement a été réalisé dans 47 cas (78%) par des sages-femmes, 11 cas (19%) par un gynécologue obstétricien et 2 cas (3%) par une matrone. Tous les enfants sont nés à terme. Le poids moyen de naissance était de 3604g [2150g et 4500g]. Dix-sept des nouveau-nés (28%) avaient une macrosomie (poids ≥ 4000g). Le membre supérieur droit était atteint dans 40 cas (67%) et le gauche dans 20 cas (33%). Il a été noté 47cas (78%) de paralysie du groupe I de Narakas suivi de celles du groupe II (n=7; 12%) et du groupe III avec paralysie complète flasque sans syndrome d´Horner (n=6; 10%). L´immobilisation coude au corps a été initiale et associée à la rééducation chez 51 enfants (85%) contre 9 (15%) conduits immédiatement en rééducation. La récupération fonctionnelle du membre lésé a été complète chez 50 enfants (83%) après un recul de 6 mois. Le délai de récupération complète était de six semaines dans 28 cas (47%), sept à 12 semaines dans 15 cas (25%) et plus de 12 semaines 7 cas (11%). Il s´agissait de tous les patients du groupe I et trois patients du groupe II de Narakas. Dix patients (17%) avaient présenté des séquelles à type de raideur de l´épaule (n=3;5%) et d´inégalité de longueur du membre supérieur (n=7;12%).

**Tableau 2:** répartition des caractéristiques épidémiologiques des mères

Caractéristiques	Effectif	Pourcentage (%)
**Parité de la mère**		
Multipare	56	94
Primipare	3	5
Nullipare	1	1
**Lieu d’accouchement**		
Centre de santé	58	97
Domicile	2	3
**Mode d´accouchement**		
Voie basse	57	95
Césarienne	3	5
**Présentation**		
Céphalique	57	95
Siège	3	5
**Difficulté obstétricale**		
Eutocique	44	74
Dystocique	14	23
Non précisé	2	3

## Discussion

L´objectif de cette étude était de décrire les aspects épidémiologiques, thérapeutiques et évolutifs de la paralysie obstétricale du plexus brachial dans notre unité de chirurgie pédiatrique. Cette présente étude a révélé que cette affection survenait chez des enfants, nés de mères multipares ayant accouché par voie basse dans un centre santé le plus souvent. Le groupe I de Narakas prédominait et l´immobilisation coude au corps associée à la rééducation fonctionnelle ont donné des résultats fonctionnels satisfaisants dans la majorité des cas. Les limites de cette étude sont liées essentiellement à son caractère rétrospectif, aux biais de sélection et à la prise en charge thérapeutique limitée au bandage coude au corps et la rééducation compte tenu de l´absence de la microchirurgie nerveuse dans notre hôpital. Cependant, Même si les résultats ne montrent pas les statistiques nationales, cette étude pourrait constituer une base de données pouvant servir de référence pour les futurs travaux sur les POPB. La paralysie obstétricale du nouveau-né n´a pas disparu malgré les importants progrès de l´obstétrique. Il n´existerait pas de facteurs de prédiction [6]. La fréquence dans cette étude était comparable à celle des séries de Tchagbele *et al*. [14] à Lomé (33,9%), de Nandjui *et al*. [15] à Abidjan (0,4%) et de Gilbert [16] en Californie (1,5%). La fréquence de cette affection est variable d´une série à l´autre. La césarienne aurait un effet protecteur important qui entraînerait une incidence plus faible mais n'empêcherait nullement la survenue de POPB [4,17,18]. Dans cette étude, la majorité des enfants était née par voie basse. Il s´agit en effet de la voie d´accouchement la plus pratiquée dans notre contexte, vu que peu de centres de santé périphériques disposent de bloc opératoire. La majorité des enfants a été vu en consultation en moyenne à une semaine de vie. Très souvent dans notre pratique, ce sont les parents qui constatent après leur sortie de l´hôpital, une hypo mobilité du membre de l´enfant. Le diagnostic du POPB devrait pourtant se faire en salle d´accouchement [10]. Il faudrait une évaluation clinique complète des fonctions sensitivomotrices du nouveau-né à la naissance et demander en cas de doute un électromyogramme ou une IRM neurologique qui confirmera les lésions nerveuses ainsi que leur gravité [9]. L´examen du bébé en salle d´accouchement devrait faire appel à la vigilance de l´examinateur et devrait être répété. Dans certains cas, le diagnostic est fait en salle d´accouchement mais les parents ne viennent pas directement en consultation avec le nouveau-né pour diverses raisons évoquées: problèmes financiers, croyances culturelles, préférence du traitement traditionnel.

Les facteurs de risque de survenue de POPB relevés dans la littérature ont été observés dans cette présente étude: il s´agit principalement de la macrosomie et de la dystocie de l´épaule chez le nouveau-né [1,2,8,19]. Les facteurs maternels les plus importants dans la littérature que sont le diabète gravidique et la prise de poids excessive [20] pendant la grossesse n´ont pas été observés. Toutefois, la multiparité observée dans cette étude est un facteur favorisant relevé par plusieurs auteurs [21,22]. Le tableau clinique était dominé par la paralysie de type Duchenne-Erb (type I de Narakas). C´est la forme de POPB la plus fréquente [9,12,14-16]. Ces lésions hautes (C5-C6) gardent classiquement un potentiel évolutif favorable au plan de la régression des lésions nerveuses [12,15,23,24]. L´immobilisation coude au corps et la rééducation fonctionnelle reste la seule alternative thérapeutique dans notre pratique. La chirurgie devrait en principe être indiquée chez certains patients qui n´avaient pas récupéré la fonction motrice après plus de 3 mois de kinésithérapie thérapie ou qui avaient des lésions graves à l´électromyogramme. Cependant, il n´existe pas d´électromyographie ni d´équipe chirurgicale qualifiée pour la chirurgie de la POPB dans notre hôpital. Il convient toutefois de souligner que selon certains auteurs, la rééducation constitue l´essentiel du traitement avec 50 à 80% de récupération fonctionnelle en 6 mois [12,23]. A contrario, d´autres soutiennent que les résultats de la chirurgie étaient supérieurs à ceux du traitement non opératoire [13,14,16]. Malgré nos possibilités thérapeutiques limitées, l´évolution des POPB a été favorable avec une récupération fonctionnelle complète dans la majorité des cas. Alloh *et al*. [12] et Tchagbele *et al*. [14] ont observé des résultats similaires. Le délai moyen de récupération observé était comparable aux données de plusieurs auteurs [12,23,25,26]. Par ailleurs, il a été noté que, certains nouveau-nés qui ont été immobilisés dans les premiers jours de vie, présentaient des signes cliniques de récupération motrice dès les premières semaines. Les enfants amenés précocement en consultation guériraient dans un délai plus court que ceux consultant tardivement [12,14]. Dans la présente étude, des séquelles ont été observées à type de raideur de l´épaule et d´inégalité de longueur des membres supérieurs. Il s´agit de séquelles inesthétiques en plus de gêner la fonction du membre supérieur. Des fonctions simples telles que porter la main à la bouche ou au-dessus de la tête seront compromises du fait de la rétraction musculo-tendineuse. Le traitement de ces séquelles fait appel à des interventions chirurgicales complexes allant des transferts tendineux à des ostéotomies pour transformer au mieux la fonction et l´esthétique du membre concerné [27,28].

## Conclusion

La paralysie obstétricale du plexus brachial demeure une affection obstétricale d´actualité malgré les progrès de l´obstétrique. La paralysie haute (C5-C6) est la plus observée. Le traitement conservateur par immobilisation coude au corps suivie des séances de rééducation fonctionnelle est la seule alternative dans notre contexte. Les résultats obtenus sont bons dans la majorité des cas. Il demeure cependant le problème de la prise en charge chirurgicale initiale de ceux qui présentent des lésions nerveuses graves et qui n´ont pas récupéré. Des efforts doivent être consentis dans le sens de la formation et de la mise en place de centres spécialisés pour ces enfants.

### Etat des connaissances sur le sujet

- La POPB est une affection qui est toujours d´actualité;
- Elle passe le plus souvent inaperçue, surtout lorsque l´examen en salle d´accouchement n´est pas mené avec rigueur;
- L´évolution est en rapport avec la gravité des lésions et le délai du traitement.

### Contribution de notre étude à la connaissance

- Cette affection demeure fréquente en pratique et cette étude constitue une base de données dans notre unité; l´accent doit être mis sur l´examen du nouveau-né en salle d´accouchement;
- Les formes graves ne peuvent être traitées chirurgicalement dans notre contexte, laissant ainsi de nombreux enfants avec un handicap majeur;
- L´avenir devrait être porté sur la chirurgie de la POPB; cela doit passer par la formation et la mise en place de centres spécialisés.

## References

[1] Foad SL, Mehlman CT, Ying J (2008). The epidemiology of neonatal brachial plexus palsy in the United States. J Bone Joint Surg Am.

[2] Bacle B, Magnussen EB, Johansen OJ, Sellaeg G, Russwurn H (2008). Obstetric brachial plexus palsy: a birth injury not explained by the known risk factors. Acta Obstet Gynecol Scand.

[3] Sibinski M, Synder M (2007). Obstetric brachial plexus palsy-risk factors and predictors. Ortop Traumatol Rehabil.

[4] Suneet PC, Sean BB, Cande VA (2014). Neonatal brachial plexus palsy: incidence, prevalence and temporal trend. Semin Perinatol.

[5] Hammad IA, Chauhan SP, Gherman RB, Ouzounian JG, Hill JB, Abuhamad AZ (2013). Neonatal brachial plexus palsy with vaginal birth after cesarean delivery: a case-control study. Am J Obstet Gynecol.

[6] Simona Z, Francesco PB, Fabrizio S, Nicola F, Vito B, Giovanni B (2018). Obstetric brachial plexus palsy : a population-based retrospective case-control study and medicolegal considerations. J Matern Fetal Neonatal Med.

[7] Chauhan SP, Christian B, Gherman RB, Magann EF, Kaluser CK, Morrison JC (2007). Shoulder dystocia without versus with brachial plexus injury: a case-control study. J Matern Fetal Neonatal Med.

[8] Stergios KD, Sabaratnam A (2010). Is it possible to reduce obstetrical plexus palsy by optimal management of shoulder dystocia?. AnnNY Acad Sci.

[9] Kouamé DB, Kouamé YGS, Sounkéré M, Koffi M, Boka ER, Odehouri TH (2014). Résultats du traitement non opératoire des paralysies obstétricales du plexus brachial (POPB) au CHU de Yopougon: Abidjan Côte d´Ivoire. J Pediat Puréicult.

[10] Lütschg J (2019). Paralysie obstétricale du plexus brachial: que faire?. Forum Med Suisse.

[11] Gary Mc Abee N, Carman C (2006). Medical and legal issues related to brachial plexus injuries in neonates. Jam Osteopath Assoc.

[12] Alloh D, Nandjui B, Manou B, Datie A, Bombo J, Anoumouye N (2005). Paralysie obstétricale du plexus brachial: résultat fonctionnel après kinésithérapie. J Réadapt Med.

[13] Egloff DV, Narakas AO (1991). Microsurgical emergencies with the exception of reinplantations. Ann Chir Main Memb Super.

[14] Tchagbele OB, Segbedji KAR, Belo M, Minoungou BM, Guedenon JK, Azoumah KD (2013). Paralysie obstétricale du plexus brachial: aspects épidémiologique et thérapeutique à propos de 65 cas colligés en trois ans au CHU Sylvanius Olympio de Lomé (Togo). J Pediatr Pueri.

[15] Nandjui B, Guedegbe F, Datie A, Kone B, Waota AB (1995). Épidémiologie et rééducation de la paralysie obstétricale du plexus brachial: à propos de 66 cas. J Réadapt Med.

[16] Gilbert A (2009). Conduite à tenir et résultats du traitement de la paralysie obstétricale du nouveau-né. Neurochirurgie.

[17] Israel Alfonso, Gemma Diaz-Arca, Daniel Alfonso T, Hans H Shuhaiber, Oscar Papazian, Andrew Price E (2006). Fetal deformations: a risk factor for obstetrical brachial plexus palsy?. Pediatr Neurol.

[18] Al-Qattan MM, el-Sayed AA, al-Kharfy TM, al-Jurayyan NA (1996). Obstetrical brachial plexus injury in newborn babies delivered by caesarean section. J Hand Surg Br.

[19] Aaron Caughey, Per Sandberg L, Marya Zlatnik G, Marie-Paule Thiet, Julian Parer T, Russell Laros K (2005). Forceps compared with vacuum: rates of neonatal and maternal morbidity. Obstet Gynecol.

[20] Alfonso DT (2011). Causes of Neonatal Brachial Plexus Palsy. Bull NYU Hosp Jt Dis.

[21] Soni L, Mir A, Kishan J, Faquih M, Elzouki Y (1985). Brachial plexus injuries in babies born in hospital: an appraisal of risk factors in a developing country. Ann Trop Paediatr.

[22] Moragianni VA, Hacker MR, Craparo FJ (2012). The impact of length of second stage of labor on shoulder dystocia outcomes, a retrospective cohort study. J Perinat Med.

[23] Courtivron B, Bonnard C, Glorion B (1993). Les paralysies obstétricales du plexus brachial: stratégie actuelle de prise en charge. Rev Méd Tours.

[24] Bisinella GL, Birch R (2003). Obstetric brachial plexus lesions: a study of 74 children. registered with the British pediatric surveillance unit J Hand Surg.

[25] Glibert A, Dumontier C (1989). Paralysie obstétricale: évolution et traitement chirurgical. Rev Prati.

[26] Davidsen M, Hvid I, Møller-Madsen B (2005). Primary treat-ment of Duchenne-Erb/Klumpke´s paralysis. Ugeskr Laeger.

[27] Nicholas Smith C, Peter Rowan, Laurel Benson J, Marybeth Ezaki, Peter Carter R (2004). Neonatal brachial plexus palsy: outcome of absent biceps function at three months of age. J Bone Joint Surg Am.

[28] Nath RK, Somasundaram C (2013). Extended long-term (5 years) outcomes of triangle tilt surgery in obstetrichial plexus injury. Open Orthop J.

